# Knock-Out of ACY-1 Like Gene in *Spodoptera litura* Supports the Notion that FACs Improve Nitrogen Metabolism

**DOI:** 10.1007/s10886-024-01512-y

**Published:** 2024-06-24

**Authors:** Tsuyoshi Maruoka, Yu Shirai, Takaaki Daimon, Rei Fujii, Masako Dannoura, Irmgard Seidl-Adams, Naoki Mori, Naoko Yoshinaga

**Affiliations:** 1 Division of Applied Life Sciences, Graduate School of Agriculture, Kyoto University, Sakyo, Kyoto 606-8502 Japan; 2 Independent Researcher, Philadelphia, PA USA

**Keywords:** FACs, Hydrolase, Lepidoptera, CRISPR/Cas9

## Abstract

Volicitin [*N*-(17-hydroxylinolenoyl)-L-glutamine] and *N*-linolenoyl-L-glutamine were originally identified in the regurgitant of *Spodoptera exigua* larvae. These fatty acid amino acid conjugates (FACs) are known to be elicitors that induce plants to release volatile compounds which in turn attract natural enemies of the larvae such as parasitic wasps. FAC concentrations are regulated by enzymatic biosynthesis and hydrolysis in the intestine of Lepidoptera larvae. It has been proposed that FAC metabolism activates glutamine synthetase and plays an important role in nitrogen metabolism in larvae. In this study, we identified candidate genes encoding a FACs hydrolase in *Spodoptera litura* using genomic information of various related lepidopteran species in which FACs hydrolases have been reported. We analyzed the importance of FAC hydrolysis on caterpillar performance with CRISPR/Cas9 knock outs. Larvae of strains with an inactive FACs hydrolase excreted FACs in their feces. They absorbed 30% less nitrogen from the diet compared to WT caterpillars resulting in a reduction of their body weight of up to 40% compared to wild type caterpillars. These results suggest that the hydrolysis of FACs is an important metabolism for insects and that FACs are important for larval growth.

## Introduction

Many plants respond to herbivory with a release of volatile organic compounds (VOCs), which is called inducible defense in contrast to constitutive defense such as a strong cell wall or the release of stored toxic components. To respond with an inducible defense response, plants need to detect feeding damage and distinguish it from arbitrary wounding events. Plants have evolved recognition systems of various substances that they are exposed to specifically during feeding damage. Such a substance that elicits the defense system of plants is called an elicitor.

One class of elicitors are the well-known FACs (Fatty acid Amino acid Conjugates), which are present in the oral secretion of several lepidopteran larvae feeding on plants (Yoshinaga et al. 2010). They induce the release of volatile organic compounds by plants in response to feeding damage (Turlings et al. 1993; Alborn et al. 1997). FACs are compounds in which fatty acids are conjugated with an amino acid. Two-thirds of the lepidopteran species analyzed so far had FACs (Yoshinaga et al. 2010). The fatty acid portion of FACs is a plant-derived C18 fatty acid such as linoleic acid or linolenic acid, with possible hydroxylation at the 17th and 18th positions, while the amino acid portion is limited to insect-derived glutamine or glutamic acid, and so far no other amino acid types have been found (Pare et al. 1998; Pohnert et al. 1999; Yoshinaga et al. 2010, 2014). FACs are actively produced in the insect’s gut cells and present in high concentrations in the caterpillar gut (1.3-3 nmol/µl) (Alborn et al. 2000; Mori et al. 2001; Lait et al. 2003). Since FACs are prevalent in the gut and oral secretion despite the fitness cost of inducing plant defenses, it seems these compounds must play important physiological roles in the larvae. Otherwise, over evolutionary time they should have lost compounds that act as elicitors of plant defenses without playing other functional roles. It was suggested that FACs acts as a surfactant involved in digestion and absorption, similar to bile acids in humans (Spiteller et al. 2000). If FACs only serve as surfactants, the amino acid portion of FACs could be any amino acids rather than exclusively L-glutamine and L-glutamic acid. Interestingly, no elicitor activity was detected when the amino acid portion of FACs was changed to other amino acids (Alborn et al. 1997; Sawada et al. 2006). Thus, in order to avoid inducing plant defenses, selective pressure should have resulted in other amino acid taking the place of Glutamine or Glutamic acid.

The other hypothesis is that FACs may function as a form of storage of glutamine, an important compound of nitrogen metabolism. Various experiments with ^14^C-labeled glutamine, glutamic acid, and linolenic acid revealed that FAC synthesis plays an active role in nitrogen assimilation (Yoshinaga et al. 2008). FACs synthesized in midgut cells are secreted into the intestinal tract, where they are eventually hydrolyzed to fatty acids and amino acids. By secreting FACs into the intestinal tract, the reaction for synthesizing glutamine from glutamic acid is activated. Free glutamine, both contained in diet and released from the FACs hydrolysis, can be absorbed quickly into the hemolymph. With such a system, free ammonia is efficiently incorporated into nitrogen metabolism, and increases the efficiency of glutamine synthase. The efficiency of nitrogen metabolism by FACs not only reduces exposure to toxic secondary metabolites by reducing the amount of food required, but also contributes to the rapid growth of lepidopteran larvae which consequently shorten the larval period exposed to various risks such as predation and parasitization. The balance between conjugation and hydrolysis seems to be important in the metabolic cycle of FACs, but the contribution of these compounds to the insect physiology has not been quantitatively demonstrated. To assess the effects of the metabolic cycle of FACs in insects, it is necessary to knock out FACs synthase or hydrolase, and to compare the fitness of the genetically modified strain with the wild-type strain. FACs synthases have not yet been identified but a hydrolyzing enzyme, a lepidopteran aminoacylase (*L-ACY-1*), has been identified in some lepidoptera, *Heliothis virescens*, *Helicoverpa zea*, *Heliothis subflexa* (Kuhns et al. 2012). Since the genomic information of *Spodoptera litura* is accessible in the International Nucleotide Sequence Database (Cheng et al. 2017), we identified the ortholog of *L-ACY-1* in *S. litura* (*Sl-ACY-1*) and knocked it out with the CRISPR/Cas9 system. We then analyzed changes in metabolism due to the deficiency in FACs-degrading enzyme ability and inferred the physiological role of FACs.

## Materials and Methods

### Insects

Wild-type (WT) strain of *Spodoptera litura* (Lepidoptera: Noctuidae) was obtained from Ishihara Sangyo Co., Ltd. Larvae were reared on an artificial diet (Insecta-LFS, Nihon Nosan Kogyo Ltd.) at 24 ± 1 °C, 50 ± 10% RH, and 16 L/8D photoperiod. Pupae were sexed, and adults were supplied with 10% honey solutions.

### Phylogenetic Sequence Analysis

The search for putative *Sl-ACY-1* genes was conducted using the fully assembled genome sequences of *S. litura* via BLAST. *H.virescens L-ACY-1* (JF922295) was submitted as a query sequence. One gene (XP02281) annotated as aminoacylase-1 A like was found highly homologous (query coverage: 99%, percent identity: 72.62%) to *H. virescens L-ACY-1*. Amino acid sequences of *Sl-ACY-1*(XP02281) and *L-ACY-1* from other lepidopteran species registered in the database were aligned using Clustal_W. A phylogenetic tree was made by the Maximum-Likelihood method using MEGA_X software. The reliability of the branch was evaluated by bootstrap analysis (1000 replicates).

### Target Design and in vitro Synthesis of Single Guide RNA (sgRNA)

Two DNA fragments within exon 2 and exon 3 of the putative *Sl-ACY-1* (LOC111348608) were selected as the target sites, according to the criteria: 5’-GG-(N)18-NGG-3’. The sgRNA was designed to contain a T7 promoter, the target site and the guideRNA (gRNA) sequences. In the target site sequence, the protospacer adjacent motif (PAM) sequence NGG at the 3’ end was removed, and the 5’GG dinucleotide overlapped with the T7 promoter. Table 1 lists the oligonucleotide sequences for each sgRNA. These gene-specific sgRNAs were prepared with a GeneArt Precision gRNA Synthesis Kit (Thermo Fisher Scientific) according to the manufacturer’s protocol. Synthesized sgRNAs were stored at -80 °C until use.

**Table 1:** Sequence of oligonucleotide for sgRNA and Primers for genomic PCR

sgRNAs/primers		Sequences
sgRNAs	Target 1	TAATACGACTCACTATAGCTGCAGCTGCGAGTC
		TTCTAGCTCTAAAACAGCTGACTCGCAGCTGCAG
	Target 2	TAATACGACTCACTATAGTTGAACTCGCACATG
		TTCTAGCTCTAAAACCATCCATGTGCGAGTTCAA
Primers	Fw	CTATCGCCCCGTTCTAGTATATCTTC
	Rv	GCATTACCATAGGGGAAAGGTGG

### Cas9/sgRNA Ribonucleoprotein Microinjection

Fertilized eggs were collected within 2 h after oviposition, and microinjection was performed within 1 h after egg collection. Cas9 protein (Integrated DNA Technologies, cat#1,081,058) and sgRNAs (sgRNA1 + sgRNA2) were mixed and incubated at room temperature for 10–15 min. The injection solution contained Cas9 nuclease and sgRNAs at final concentrations of 1 µg/µL and 150 ng/µL respectively. Eggs were affixed onto a glass slide with double-sided adhesive tape and injected using Femtojet 4i (Eppendorf). Injected eggs were maintained at 25 °C ± 1 °C until hatching.

### Genotyping and Establishment of a Homozygous Knockout line

Genotyping of larvae and adults were carried out to identify *Sl-ACY-1* mutant alleles induced by the Cas9/sgRNA injection. For genotyping, whole neonate larval bodies and adult moth heads were homogenized with 50 mM NaOH and then heated at 95 °C for 15 min. After neutralization with 200 mM Tris-HCl (pH 8.0), the supernatants were used as the PCR templates. PCR was conducted with the primers listed in Table 1 using Green Master Mix (Promega). PCR products were analyzed with 1.5% agarose gel in 1× TAE buffer using a Mupid-2plus System (Takara Bio) at 100 V for 30 min at room temperature. Gels were submerged in 1×TAE buffer containing 0.05 µg/ml of ethidium bromide for 20 min. Gels were documented on a bioimaging system. All putative mutations were confirmed by Sanger sequencing.

To establish a homozygous knockout line, larvae hatched from injected eggs (generation zero, G_0_) were reared to adulthood and crossed with wild type (WT) moths. In the next generation (G_1_), G_1_ adults were crossed with WT, and the resulting G_2_ individuals were individually genotyped by PCR and Sanger sequencing. G_2_ moths carrying the mutation were sib-crossed and the mutation was genetically fixed in the next generation (G_3_). The resulting knockout line was maintained as a homozygous stock.

### In vitro Enzyme Activity Assay

*N*-Linolenoyl-L-glutamine for in vitro enzyme assay was synthesized as described previously (Koch et al. 1999). To isolate the enzyme, we immediately collected fresh excrement from fifth-instar larvae. The excrement was suspended in distilled water (100 mg/ 2 ml) and stored at 4 °C until used as crude enzyme extract. Enzyme activity was assayed by incubating synthetic 50 mM *N*-linolenoyl-L-glutamine (substrate FACs) in 25 mM Tris HCl buffer (pH8.0, total volume 1500 µl) with the crude enzyme solution at a final substrate concentration of 250 µM. The mixture was incubated at 37 °C for 0, 30, 60, 90, 120, 150 min. To stop the enzyme reaction each fraction was heated to 90 °C. Enzymatic degradation activity was evaluated using LCMS analysis by measuring the amount of *N*-linolenoyl-L-glutamine over time relative to the concentration of *N*-linolenoyl-L-glutamine in the intestinal tract at the start of the experiment.

### Simultaneous Quantification Nitrogen Assimilation

About 1 g of 5th instar larvae frass was collected within a certain time period and the weight of artificial diet consumed within the same time period was measured. Frass and diet samples (the same amount that was consumed) were oven-dried at 60 °C (dry weight: tens of milligrams), and thoroughly ground in a mortar. The amount of nitrogen in each sample was measured by using element analyzer (JM1000CN; J-Science Lab Co. Ltd., Kyoto, Japan). Nitrogen amounts in the diet (N_diet_) and frass (N_frass_) was calculated using the calibration curve, prepared with hippuric acid as a standard. Nitrogen assimilation ratio (NAR) was calculated below based on the percentage of the nitrogen in the ingested diet remaining in the body without being discharged in the frass (N_frass_).

$${NAR}_{(individual)}=(N_{diet}-N_{frass})/N_{diet}$$

The average nitrogen absorption ratio NAR was thus calculated as the average individual NARs for several larvae (*n* = 5).

### LCMS Analysis

Mass spectral measurements were carried out with LCMS-2010 instrument (Shimadzu). A portion of the suspended frass was injected into a reversed-phase column (Mightysil RP-18 GP 50 × 2.0 mm I.D., Kanto chemical Co., Inc.), eluted for 13 min at 0.2 ml/min with a solvent gradient of 42–98% CH_3_CN containing 0.08% acetic acid, in water containing 0.05% acetic acid. The column temperature was maintained at 40 °C, and the column eluent was monitored by continuous MS total ion current trace. The curve dissolution line (CDL) temperature was 250 °C, the voltage was 1.5 kV, the nebulizer gas flow was 1.5 l/min, and the analytical mode was ESI negative scan. The negative ionization mass spectra gave characteristic [M-H]^−^ ions for *N*-linolenoyl-L-glutamine at m/z 405, and *N*-linoleoyl-L-glutamine at m/z 407.

## Results

### Phylogenetic Analysis of ***S. Litura L-ACY-1*** Genes

**Fig. 1:**
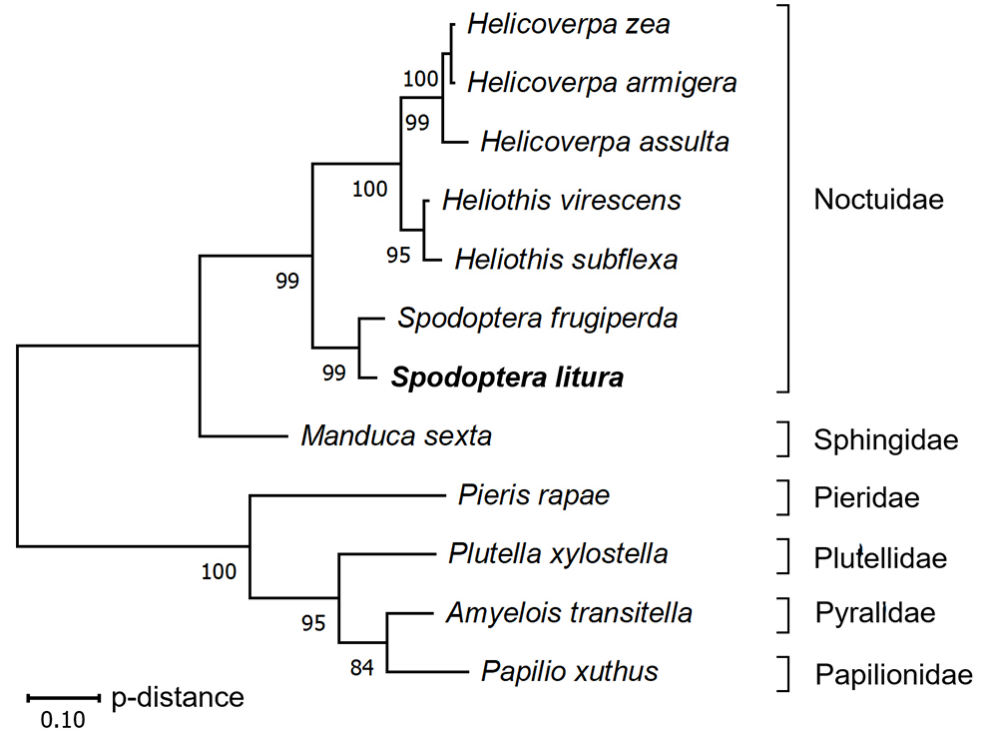
Phylogenetic tree of *L-ACY-1* gene based on amino acid sequences. Phylogenetic tree was constructed using the maximum - likelihood method using MEGAX software with 1000 replicates. The scale bar indicates the evolutionary distance. The numbers at each tree node are the bootstrap values. The accession numbers for each are as follows. *Helicoverpa zea*, AET43035; *Helicoverpa armigera*, AET43036; *Helicoverpa assulta*, AQU43265; *Heliothis virescens*, AET43034; *Heliothis subflexa*, AET43037; *Spodoptera frugiperda*, AET43033; *Spodoptera litura*, XP_022815042; *Manduca sexta*, XP_030032986; *Pieris rapae*, XP_022115118; *Plutella xylostella*, XP_048480360; *Amyelois transitella*, XP_013187319; *Papilio xuthus*, KPI96806.

The putative amino acid sequence of *S.litura L-ACY-1* was obtained through a BLAST homology search using *H.virescens L-ACY-1* (AET43034) as the query. The deduced *S. litura L-ACY-1* protein shares 93% amino acid identity with *S. frugiperda L-ACY-1*, 72% with *H. zea L-ACY-1*, 70% with *H. armigera L-ACY-1*, 70% with *H. assulta L-ACY-1*, 74% with *H. virescens L-ACY-1*, 77% with *H.subflexa L-ACY-1*, 63% with *Manduca sexta L-ACY-1*, 53% with *Plutella xylostella L-ACY-1*, 40% with *Papilio xuthus L-ACY-1*, 37% with *Pieris rapae L-ACY-1*, 39% with *Amyelois transitella L-ACY-1*. The phylogenetic tree showed that the *L-ACY-1* genes are highly conserved among the Noctuidae species. *S. litura L-ACY-1* was closest to *S. frugiperda L-ACY-1* and clustered with *L-ACY-1* genes of other Noctuidae species, suggesting conserved function (Fig. 1).

### CRISPR/Cas9-Mediated Targeted Gene Mutagenesis of the ***Sl-ACY-1***

To disrupt the *Sl-ACY-1* gene, we designed two different sgRNAs for the CRISPR/Cas9 system as shown in Table 1. 150 eggs were injected with Cas9 nuclease and a mixture of two sgRNAs at final concentrations of 1 µg/µL and 150 ng/µL, respectively. Ten larvae hatched and six of them survived to adulthood. No obvious phenotypic changes were observed in these G_0_ insects. The G_0_ adults were individually crossed with WT to see if the induced mutations could be inherited by the next generation (G_1_). G_1_ larvae were hatched from three egg batches, and we randomly selected eight first instar G_1_ larvae from each batch to examine their genotype using genomic PCR and Sanger sequencing. Of the three batches, one batch contained gene-edited individuals (3 larvae out of 8 examined). All these three G_1_ larvae had the same large deletion alelle (i.e., 487-bp deletion and 1-bp insertion) flanked by the two sgRNA target sites.

### Generation of the ***Sl-ACY-1*** Mutant Strain

**Fig. 2:**
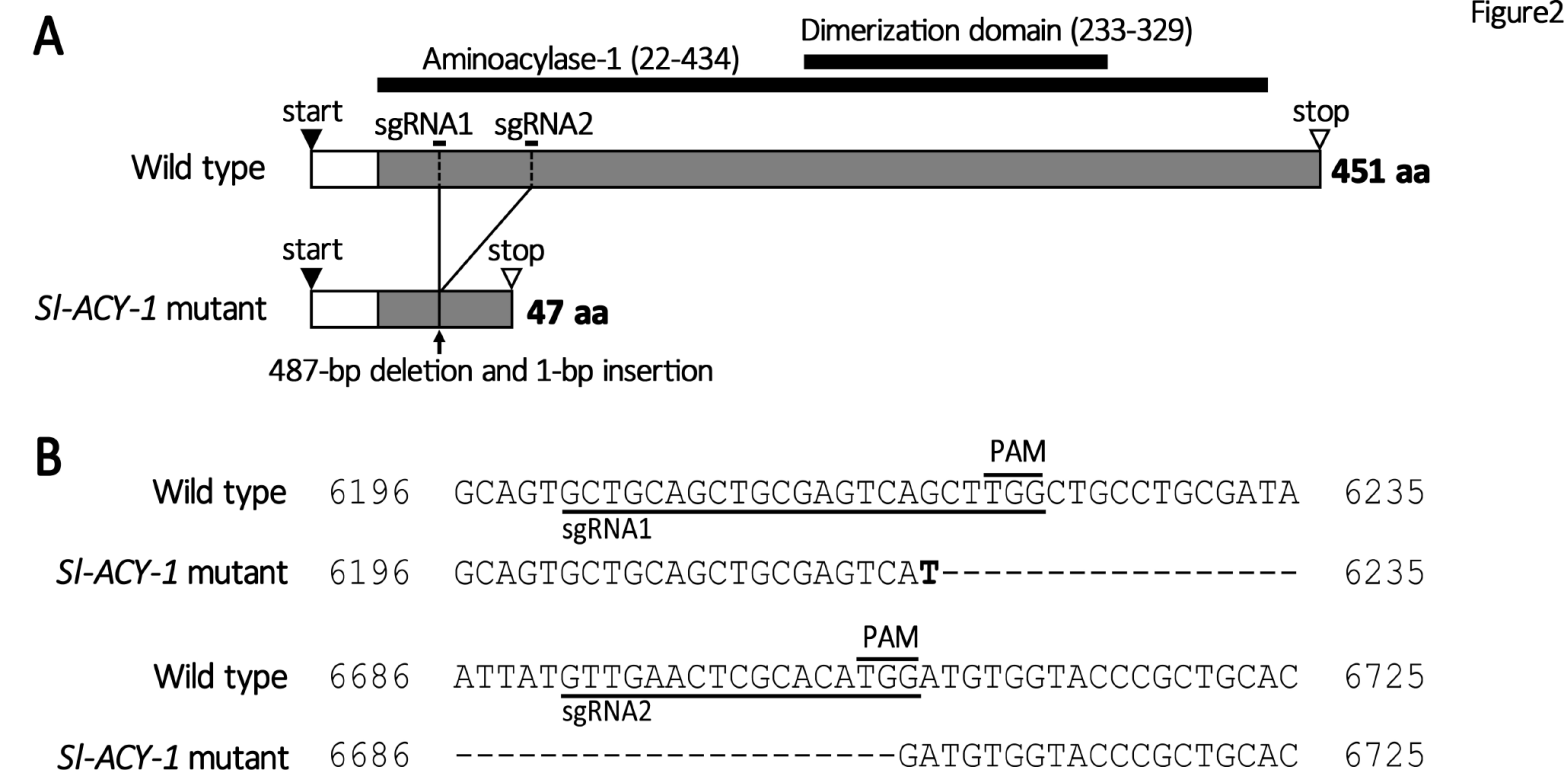
(A) The comparison of CDS regions between wild type (XM_022959274.1) and *Sl-ACY-1* mutant. Closed and open arrowheads indicate start and stop codons, respectively. *Sl-ACY-1* mutant strain has a 487-bp deletion and a 1-bp insertion (resulting in a net loss of 486-bp), and a new stop codon is created midway. Positions of the aminoacylase and the dimerization domain are indicated by black lines. Signal peptide region is abbreviated as “SP.” (B) The nucleotide sequence of Spodoptera litura. The numbers indicate the positions on the scaffold LOC111348608. CRISPR/Cas9 caused a deletion of 487-bp, with a T (highlighted in bold) inserted

To genetically fix the mutant allele of *Sl-ACY-1*, G_1_ adults were individually crossed with WT. The resulting G_2_ were then genotyped after hatching. The G_2_ adults carrying the defined mutation (i.e., 487-bp deletion and 1-bp insertion; see Fig. 2) were sib-crossed to generate homozygous individuals in G_3_. The resulting homozygous adults were used to establish the *Sl-ACY-1* knockout line of *S. litura*, which was maintained as a homozygous line for > 15 generations.

### Phenotypes of ***Sl-ACY-1*** Mutant

**Fig. 3:**
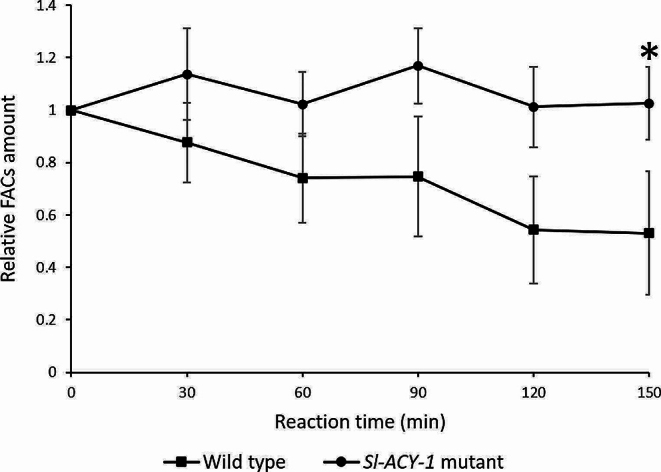
Changes over time in FACs due to enzymatic reactions. The amount of FACs at the beginning of the reaction was set as 1, and the relative amounts are shown over time. In the wild type, the graph shows a decrease over time, whereas in the *Sl-ACY-1* mutant, the graph is flat. This means that the FACs were not degraded in the *Sl-ACY-1* mutant. Significant difference is shown by asterisk, *n* = 4, mean ± SD, Welch’s *t*-test, *p* < 0.05

To test whether *Sl-ACY-1* mutant *S. litura* lost the hydrolytic potential of FACs, an enzyme assay for FACs hydrolytic activity was conducted with WT and *Sl-ACY-1* mutant frass. Enzyme activity was measured by quantifying by LCMS the remaining amount of the substrate *N*-Linolenoyl-L-glutamine (NLLG) over time. As shown in Fig. 3, the crude enzyme solution from WT caterpillars showed continued hydrolysis of NLLG over time, whereas no NLLG decreases were detected with enzyme preparations from *Sl-ACY-1* mutant caterpillar frass, suggesting that the mutant lost the capability to hydrolyze FACs. Therefore, we concluded that *Sl-ACY-1* is in fact a hydrolase of FACs in *S. litura*.

### FACs Analysis of Frass

**Fig. 4:**
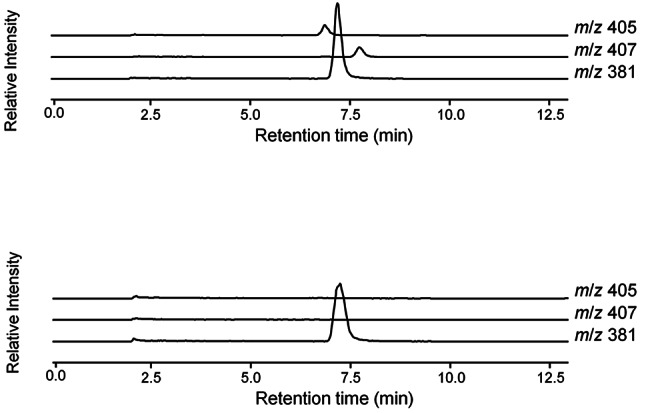
LCMS analysis for detection of FACs in frass. A peak indicating FACs was detected in the chromatogram of the mutant, but not in that of the wild type *N*-palmitoleoyl-L-glutamine: *m*/*z* 381 (Internal standard), *N*-linoleoyl-L-glutamine: *m*/*z* 407, *N*-linolenoyl-L-glutamine: *m*/*z* 405

The in vivo activity of *Sl-ACY-1* in *S. litura* larvae was evaluated by analyzing the FACs concentration in the frass. Since the negative impact of the gene mutation may be covered by unnaturally rich nutrient content as found in artificial diet, the assay was conducted using artificial diet diluted with agar to mimic the nutrient content of natural plant tissues. 5th instar WT larvae fed on this modified artificial diet excreted no FACs in the excrement as previously observed, when they fed on plant leaves. *Sl-ACY-1* mutant larvae excreted detectable amounts of FACs as shown in Fig. 4. WT *S. litura* 4th instar larvae hydrolyzed *N*-linolenoyl-L-glutamine gradually, whereas *Sl-ACY-1* mutant larvae did not. Therefore, we concluded that *Sl-ACY-1* mutant larvae lost their capability to hydrolyze FACs.

Both experiments show that *Sl-ACY-1* is indeed a FACs hydrolase: crude enzyme preparations from *Sl-ACY-1* mutant larval frass showed no FACs hydrolytic activity in an invitro enzyme activity test, and when feeding on a nutrient limited diet *Sl-ACY-1* mutant caterpillars excreted FACs while WT caterpillars did not. Supporting the hypothesis that the mutants do not have a functioning FACs hydrolase.

*N*-palmitoleoyl-L-glutamine: *m*/*z* 381 (Internal standard), *N*-linoleoyl-L-glutamine: *m*/*z* 407, *N*-linolenoyl-L-glutamine: *m*/*z* 405.

### Simultaneous Quantification of Carbon and Nitrogen in Excrement

**Fig. 5:**
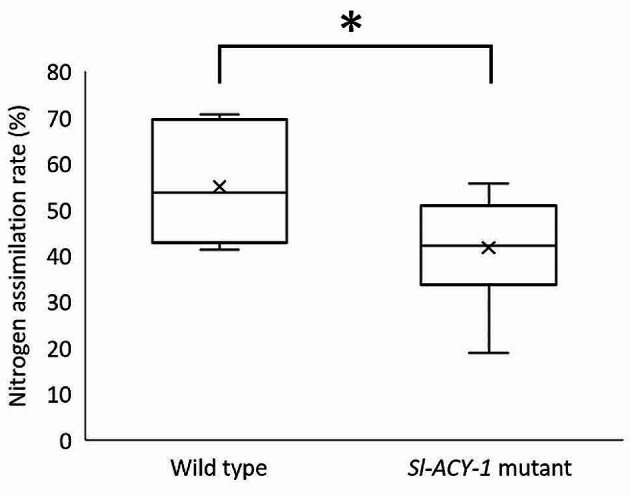
Nitrogen assimilation assay (*n* = 5, respectively). Nitrogen concentration was higher in the frass of *Sl-ACY-1* mutant than that of wild type. Consequently, the nitrogen assimilation efficiency was significantly lower in *Sl-ACY-1* mutant. Significant difference is shown by asterisk, *p* < 0.05, Wilcoxon rank sum test

When 2nd instar caterpillars fed on a nutrient limited diet the nitrogen absorption rate of *Sl-ACY-1* mutant caterpillars was about 70% of that of the wild type caterpillars (Fig. 5). That is, *Sl-ACY-1* mutant caterpillars absorbed nitrogen less efficiently presumably because FACs cannot be hydrolyzed. This fact indicates that the loss of nitrogen caused by excretion of FACs accounts for almost 30% of total nitrogen absorption. In other words, wild type *S. litura* caterpillars usually absorb 30% of the nitrogen intake through the hydrolysis of FACs within the intestine.

### Growth Measurement of ***S. litura***

**Fig. 6:**
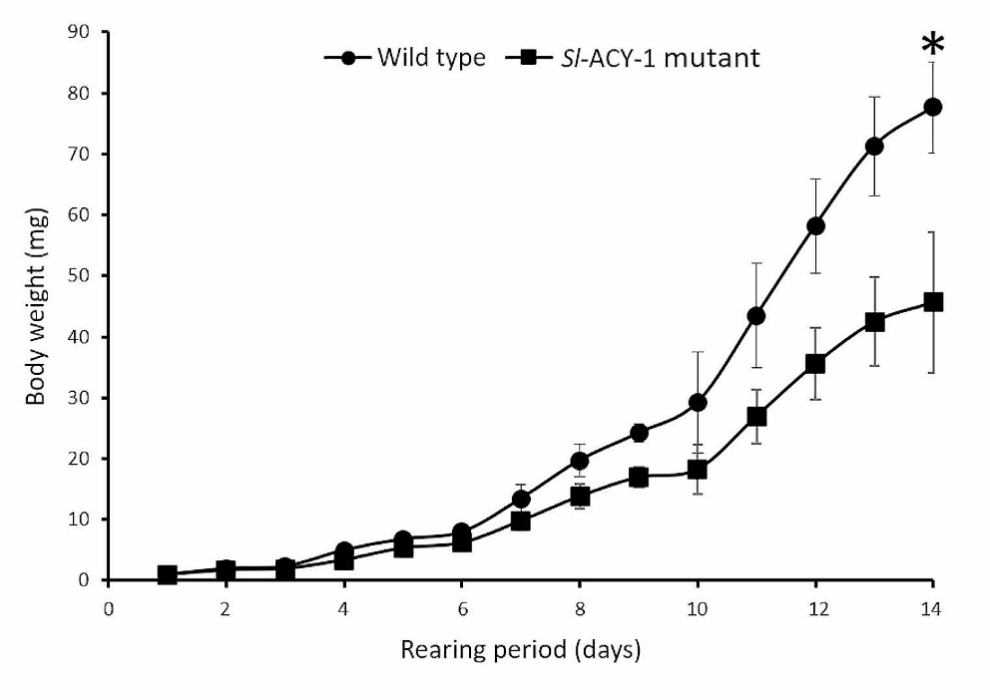
Comparison of the growth curve (*n* = 6, respectively. Mean ± SD, Welch’s t-test, *P < 0.05). *Sl-ACY-1* mutant grows slower than wild type. On day 14, *Sl-ACY-1* mutants weighed only about 60% of the wild type

To evaluate how the nitrogen loss caused by FACs excretion may impact larval growth, the larval weight was recorded from 2nd instar to last instar. *Sl-ACY-1* mutant larvae grew slower than wild type. The difference was the largest on the 14th day and the body weight of the mutant strain was about 60% of that of wild type (Fig. 6). Most WT larvae pupated after 29 days while the mutant larvae showed a few days delayed pupation (data not shown).

## Discussion

Since the first identification of FACs from regurgitant of *S. exigua* larvae (Alborn et al. 1997), the physiological role of FACs in the insects have been studied testing the hypothesis that FACs play an important role in nitrogen assimilation (Yoshinaga et al. 2008). Although several data supported this hypothesis, decisive evidence has been lacking. Knockout/knockdown of FACs synthase or hydrolase are required to directly see the effect of defective FACs metabolism in vivo in larvae but in order to do that several technical obstacles had to first be overcome.

*S. litura* is an infamous lepidopteran pest insect ubiquitous throughout tropical and temperate Asia. Not until 2017, was its genome sequenced, and the gene database made accessible. *S. litura* was the insect in which the FACs function was first linked to a nitrogen assimilation enhancer (Yoshinaga et al. 2008). Fortunately, CRISPR/Cas9-mediated gene editing system was recently developed for this species (Shirai et al. 2021), thus making it suitable to investigate the function of FACs. We identified in *S. litura* an ortholog of *L-ACY-1*, a hydrolase of FACs found in other lepidopteran species (Kuhns et al. 2012), and named it *Sl-ACY-1*. We succeeded in generating a knockout mutant line of *Sl-ACY-1*. We confirmed that *Sl-ACY-1* is a hydrolase of FACs of *S. litura* because the FACs hydrolysis activity was lost in the knockout larvae. Usually organisms have (small) gene families which can complement each other in an important function. While we identified two additional genes, which are highly related to one another, we were lucky that the gene which is closely related to other lepidopteran *L-ACY-1*s encodes a hydrolase and seems to be responsible for the majority if not for all of the FACs hydrolase activity in *S. litura*.

Typically, WT insects excrete only trace amounts of FACs in their frass pellets when they are feeding on plant leaves (Yoshinaga et al. 2008) but when feeding on nutrient rich artificial diet they excrete the excessively synthesized FACs (Yoshinaga, unpublished). Therefore, we developed a “diluted diet” which resulted in undetectable excretion of FACs by WT caterpillars. Yet, mutant caterpillars which were feeding on this diluted diet still excreted detectable amounts of FACs in their frass. In vitro enzyme assay and LCMS analysis of frass in this study revealed that *Sl-ACY-1* mutant caterpillars could not hydrolyze FACs and therefore excreted it in the frass. Apparently, the loss of hydrolase *Sl-ACY-1* in the mutant did not affect their FAC synthase activity and the imbalance of synthesis and hydrolysis resulted in the purge of FACs with the frass.

This loss of FACs led to less efficient nitrogen metabolism. The total nitrogen absorption rate of the mutant decreased by almost 30% of that of the WT strain (Fig. 5). This decrease was due to the high concentration of nitrogen content in the frass of mutants. This inefficiency of nitrogen metabolism resulted in the slower growth rate of the larvae. The delay of weight gain shown in mutant strains reached at maximum 40% of that of WT (Fig. 6). After day 15, individual differences widened and no significant differences between the two groups could be seen, but the mutant strain tended to have a lower rate of weight gain, which continued until the end of the larval stage. These results are the first quantitative data showing the physiological importance of FACs hydrolase in insects.

The rapid growth of these pests despite their nitrogen-poor plant diet can be partly explained by the FAC system that enhances glutamine synthesis and ammonia recycling (Yoshinaga et al. 2008). As we have shown in this study, disrupting the system results in the risk of nitrogen loss especially if the hydrolase does not work.

Given how high the contribution of FACs to the nitrogen absorption of the insect is, plants could defend themselves from gluttonous lepidopteran insects by targeting their FACs metabolism, especially since many global lepidopteran pests are known to have them (Yoshinaga et al. 2010).

Interestingly, it is reported that when larvae consumed gossypol, a toxic sesquiterpene dimer produced by cotton plants, *L-ACY-1* is inhibited in some species (Krempl et al. 2021). The authors suggested the disadvantage for the insect losing their hydrolase activity is an increase of FAC concentration in their midgut, which can elicit stronger defense responses from plants. Our finding suggests that a compromised hydrolase activity may account for an active nitrogen loss, subsequent weight loss and thus extension of larval stage (data not shown). This also benefits the natural enemies for giving them more opportunity to lay eggs or prey on the larvae.

It is not clear how serious it is for the insects if the FAC system did not work at all. Some lepidopteran insects do not have FACs, and these insects manage the nitrogen metabolism without FACs, yet they might have a different mechanism in place. Evaluating the importance of FACs for insects is not possible without a knockout study of FAC synthase in these FAC-using insects. Unfortunately, the FAC synthase is not yet identified.

Plants have evolved a sophisticated inducible defense system, which requires detecting insect feeding and distinguishing it from abiotic wounding. Thus, plants are capable of detecting insect produced elicitors. Theoretically, plants could use any compounds characteristic to the caterpillar spit as an elicitor, but it must be present consistently when the insect is feeding and it should be indispensable for the insects. The results obtained in this experiment offer a possible explanation why some plants recognize FACs and use it as a chemical signal to detect feeding damage and why some lepidopteran insects have not lost their dependence on FACs over evolutionary time. In short, the cycle of FACs biosynthesis and hydrolysis is important for nitrogen metabolism. So far, the mechanism by which this FACs cycle enhances larval nitrogen metabolism has not been fully proven. The fact that the product of glutamine synthase is the main source of the glutamine used for FACs suggests the main focus of FAC synthesis might be on delivering this glutamine to the midgut lumen (Yoshinaga et al. 2008). In other words, FACs may function to consume the glutamine to tip the equilibrium of the glutamine synthase reaction. The promotion of nutrient metabolism by FACs might deliver a great advantage for larvae, and thus, making FACs can be a strong advantage for the insects especially in nitrogen limited environments. But for this function, the glutamine moiety of FACs cannot be replaced by a different amino acid, thus using other amino acids, which would enable the insect to avoid detection by the plant and not induce a plant defense reaction and possibly longer exposure to parasitoids is not an option. From the plant’s perspective, FACs fulfill all necessary requirements of a dependable elicitor. Therefore, the nutritional advantage which FACs provide for the insect must outweigh the disadvantage of having elicited plant defenses.

Knockout of FAC synthase is necessary for further investigation of the physiological function of FACs. The growth deterioration shown in this paper may be due to the failure to recover the nitrogen nutrient value devoted to FACs biosynthesis; the loss of FACs hydrolysis does not reduce the efficiency of nitrogen nutrient use if FACs are not synthesized. Therefore, it is necessary to examine whether the simultaneous loss of FACs synthase can restore growth retardation and reduced nitrogen absorption, as observed in this experiment, to the WT level. It is then necessary to compare the metabolism of double knock-out strains compared to WT and measure the effects of having FACs on nitrogen absorption and growth.

## Data Availability

No datasets were generated or analysed during the current study.
